# Large Pleural Effusion After Transjugular Intrahepatic Portosystemic Shunt, a Rare but Deadly Complication

**DOI:** 10.7759/cureus.16759

**Published:** 2021-07-30

**Authors:** Kushang Shah, Michael X Jin, Rachel Bright

**Affiliations:** 1 Internal Medicine, Stony Brook University Hospital, Stony Brook, USA; 2 Radiology, Stony Brook University Hospital, Stony Brook, USA

**Keywords:** transjugular intrahepatic portosystemic shunt, ascites, pleural effusion, hemorrhagic shock, disseminated intravascular coagulation (dic)

## Abstract

Cirrhosis affects more than 630,000 adults globally and can lead to development of ascites. Transjugular intrahepatic portosystemic shunt (TIPS) is an alternative option for refractory ascites in patients who are ineligible or are waiting for liver transplants. However, this procedure can have serious complications. We present a case that highlights the development of a complex pleural effusion complicated by hemorrhagic shock and disseminated intravascular coagulation after TIPS in a 54-year-old man. Our case is the first to report such a complication and aims to provide awareness.

## Introduction

Cirrhosis, also known as end-stage liver disease, affects more than 630,000 adults globally [1].  It results from scarring caused by chronic liver damage leading to permanent loss of liver function. As the liver loses function and scars, portal venous pressure is increased because blood cannot as easily transverse the liver parenchyma. This results in transudate accumulating within the peritoneal cavity [2]. As more fluid accumulates within the peritoneal cavity, patients may develop spontaneous bacterial peritonitis, a condition with a 17-32% mortality rate [2,3]. 

Definitive treatment for cirrhosis-induced ascites is liver transplantation. However, only approximately half of all eligible patients on liver transplant waitlists later receive a transplant due to the low supply of donor livers [4]. Transjugular intrahepatic portosystemic shunt (TIPS) is an alternative option for refractory ascites in such patients.  TIPS is an artificial tract created under X-ray imaging within the liver to bypass the scarred liver tissue, thereby reducing the amount of back pressure and resulting ascites. However, TIPS can be associated with a variety of complications including bleeding, infection, and hepatic encephalopathy [4]. Other complications can be rare but still occur. Our case describes one such complication in a patient who underwent TIPS and subsequently developed pleural effusions.  

## Case presentation

A 54-year-old man with a history of intravenous (IV) drug and alcohol abuse, hepatitis C, decompensated cirrhosis complicated by ascites and portosystemic encephalopathy, and factor XI deficiency presented to the emergency department with two days of difficulty breathing from ascites-induced abdominal distention.  The patient had received nine therapeutic paracenteses within the past three months due to symptomatic ascites.  On this admission, the patient’s vital signs were stable. He denied fever, chills, chest pain, nausea, vomiting, diarrhea, and constipation. Physical examination revealed moderate ascites and anasarca; minimal encephalopathy was noted per West Haven criteria [5].  Chemistry and X-ray showed elevated liver enzymes and mild pulmonary vascular congestion (Table 1). Therapeutic paracentesis drained 3.7 L of fluid negative for spontaneous bacterial paracentesis and malignancy.

**Table 1:** Patient's blood count and chemistry on admission. BUN, blood urea nitrogen; ALT, alanine aminotransferase; SGPT, serum glutamic-pyruvic transaminase; AST, aspartate aminotransferase; SGOT, glutamic-oxalacetic transaminase; WBC, white blood cells; RBC, red blood cells; MCV, mean corpuscular volume; RDW, red cell distribution width; INR, international normalized ratio; PTT, partial thromboplastin time.

Chemistry	
Sodium	134 mmol/L
Potassium	3.7 mmol/L
Chloride	106 mmol/L
Bicarbonate	19 mmol/L
Glucose Level	97 mg/dL
BUN	40 mg/dL
Creatinine	1.03 mg/dL
Anion Gap	9 mmol/L
Calcium	8.3 mg/dL
Phosphorus	2.8 mg/dL
Magnesium	2.2 mg/dL
Bilirubin, Total	2.1 mg/dL
Bilirubin, Direct	1.0 mg/dL
ALT (SGPT)	64 IU/L
AST (SGOT)	108 IU/L
Alkaline Phosphatase	257 IU/L
Albumin	3.0 g/dL
Total Protein	6.5 g/dL
Lipase Level	92 IU/L
Ntpro B-Type Natriuretic Peptide	215 pg/mL
Ammonia	66 umol/L
Lactic Acid	0.6 mmol/L
Blood Count	
WBC Count	5.24 k/uL
RBC Count	2.37 M/uL
Hemoglobin	8.7 g/dL (9.1 g/dL previous hospitalization)
MCV	108.0 fL
RDW	16.7%
Platelets	53 k/uL (44 k/uL previous hospitalization)
INR	1.4
PTT	47.8

**Figure 1:**
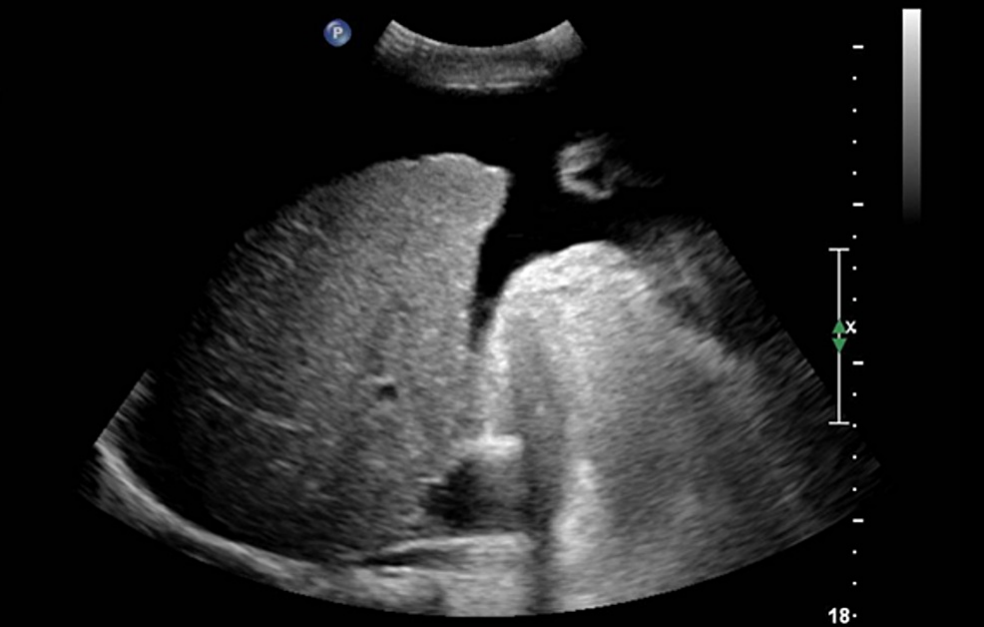
Ultrasound of right upper abdominal quadrant before transjugular intrahepatic portosystemic shunt demonstrating mild ascites.

**Figure 2:**
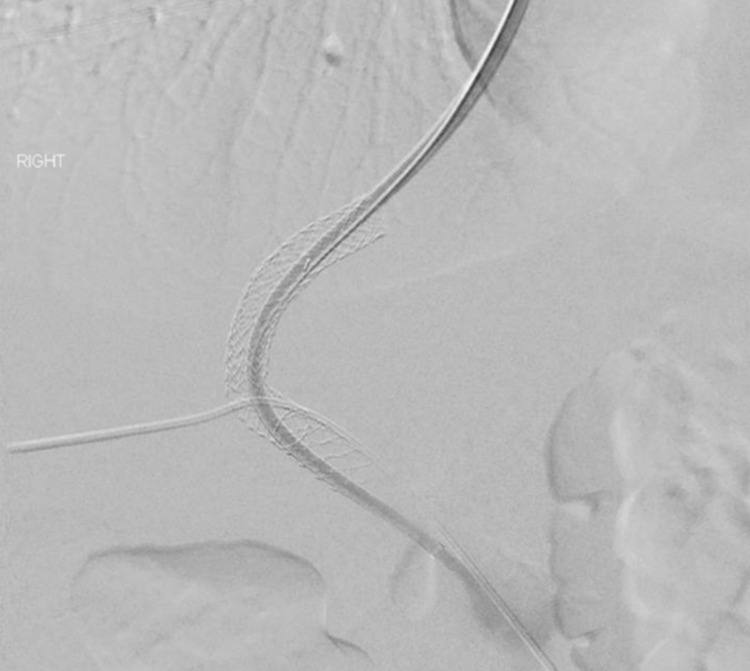
Digital subtraction angiography of the portal vein during transjugular intrahepatic portosystemic shunt placement showing successful placement of stent for procedure.

Our patient was not a candidate for liver transplant due to active IV drug use, so he elected to proceed with TIPS (Figures 1, 2). During the procedure, a firm, cirrhotic liver was noted, causing difficulties for anastomosis creation between right hepatic and portal veins. Anastomosis was eventually created with a 2 cm/6 cm Viatorr 10 mm TIPS stent and a 10 mm x 59 mm covered Viabahn extension, resulting in a portal venous gradient decrease from 11 to 1 mmHg with minimal blood loss.  

Ten hours after procedure, the patient developed confusion. Lab work revealed a glucose level of 50 mg/dL, hemoglobin of 6.7 g/dL (8.7 g/dL on admission), hyperkalemia, anion gap metabolic acidosis with respiratory alkalosis, and disseminated intravascular coagulation (DIC) (Table 2). The patient was given sodium polystyrene sulfonate, calcium gluconate, albuterol, dextrose with insulin, and one unit of packed red blood cell (pRBC). His hemoglobin dropped to 6.1 g/dL 8 hours after transfusion.

**Table 2:** Labs upon arrival to the intensive care unit. BUN, blood urea nitrogen; ALT, alanine aminotransferase; AST, aspartate aminotransferase; LDH, lactate dehydrogenase; TIBC, total iron-binding capacity; WBC, white blood cells; RBC, red blood cells; MCV, mean corpuscular volume; RDW, red cell distribution width; INR, international normalized ratio; aPTT, activated partial thromboplastin time.

Chemistry	
Sodium	135 mmol/L
Potassium	5.5 mmol/L
Chloride	109 mmol/L
Bicarbonate	17 mmol/L
Glucose Level	50 mg/dL
BUN	43 mg/dL
Creatinine	1.55 mg/dL
Anion Gap	9 mmol/L
Calcium	8.2 mg/dL
Phosphorus	4.5 mg/dL
Magnesium	2.0 mg/dL
Total Bilirubin	4.1 mg/dL
Direct Bilirubin	2.3 mg/dL
ALT	349 IU/dL
AST	953 IU/dL
LDH	742 IU/dL
Haptoglobin	10.5 mg/dL
Lipase	26 IU/L
Lactic Acid	8.0 mmol/L
Iron	102 ug/dL
TIBC	100 ug/dL
Ferritin	7877 ng/mL
B12	1773
Folate	>20
Ammonia	50 umol/mL
Troponin	0.8
Blood Count	
WBC Count	8.61 k/uL
RBC Count	1.89 M/uL
Hemoglobin	6.7 g/dL (6.1 g/dL after transfusion)
MCV	106.9 fL
RDW	19.1%
Platelet Count	74 k/uL
INR	2.1
aPTT	50.3 seconds
Arterial Blood Gas	
pH	7.29
pCO_2_	27
pO_2 _	91
HCO_3 _	12

**Figure 3:**
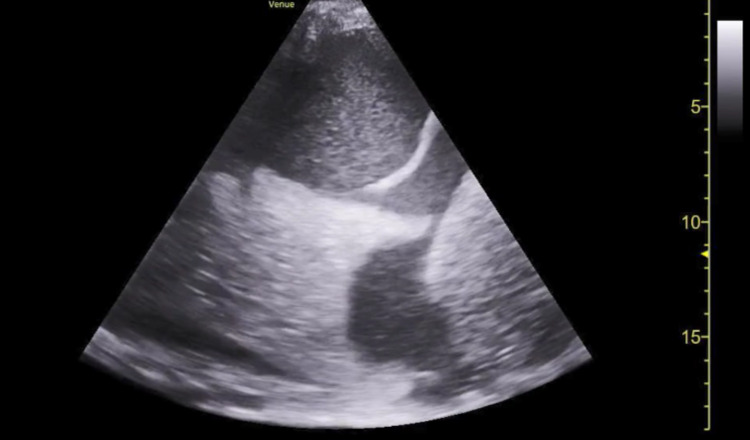
Complex right-sided pleural effusion.

Due to deteriorating clinical status, he was transferred to the medical intensive care unit (MICU).  Upon arrival, the patient was in respiratory distress. Vitals were 24 breaths per minute, temperature 34.8^o^C, heart rate 94 beats per minute, and blood pressure was 105/52 mmHg. He had grade III encephalopathy per West Haven criteria, abdominal distention, and asterixis. Right upper quadrant ultrasound revealed a large, complex right-sided pleural effusion with increased sedimentation without fluid at porta hepatis or ascites (Figure 3). Portal systemic anastomosis was patent. Cardiac ultrasound showed a hyperdynamic left ventricle without right ventricular strain. The patient was subsequently intubated. CT angiography did not show definite areas of extravasation.

Within 10 hours the patient received six additional units of pRBC, four units fresh frozen plasma, two units of platelets, and minimal norepinephrine. Diagnostic thoracentesis produced red, turbid fluid with 1,960,000 red blood cells/µL (hematocrit 21.5%), meeting criteria for hemothorax. A chest tube was placed and immediately drained 4 L of fluid. After seven days, an additional 1.47 L was drained. Repeat ultrasound showed no new effusion, and the chest tube was removed. Once our patient was hemodynamically stable, he was downgraded to medicine floors and discharged for outpatient follow-up.

## Discussion

**Figure 4:**
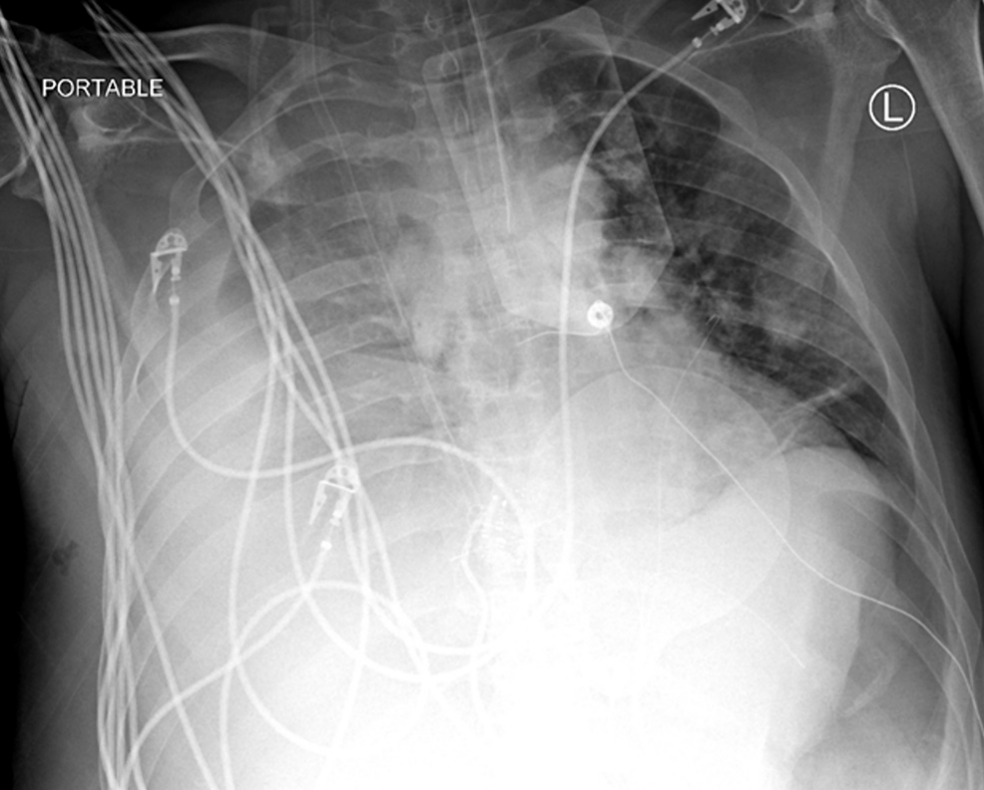
Development of overnight effusion.

Case reports regarding complications of TIPS exist; however, a variety of serious complications can occur [6,7]. Our case highlights post-TIPS development of a complex pleural effusion complicated by hemorrhagic shock and DIC. It is unclear how this effusion developed since no report exists of this complication occurring after TIPS. It is impossible that our patient’s acute chest output was 4 L of frank blood because the average human body has only 4.5-5.5 L of blood.  During the first 24 hours in the MICU, the patient’s net fluid intake totaled 2.7 L. Total output was 6.3 L, with 4.6 L from chest tube output. Had this patient exsanguinated 4 L of frank blood, even with vasopressors, he would have developed severe hemodynamic instability.  However, our patient maintained mean arterial pressure consistently >65 mmHg with minimal vasopressors. Therefore, the “hemothorax” was more likely ascitic fluid from a peritoneal-pleural communication (i.e., diaphragmatic defect) created or enlarged intraoperatively, which mixed with blood from hepatic parenchyma injury during shunt creation [7,8]. This mechanism is supported by the X-rays showing persistent pleural effusion that drained 1.47 L over one week (Figure 4). 

Alternatively, trauma to the internal jugular vein or carotid artery during the procedure could have contributed to the hemothorax.  A slowly bleeding vessel may not be immediately apparent on imaging but may collect in the pleural space over time.  A right internal jugular vein approach was used; while traversing from the internal jugular vein to the liver, any vessels in the vicinity could be injured and bleed into pleural space if a communication exists [9].  Contrary to this theory, no hematoma or acute venous bleeding was noted during the procedure.  

The risk of damage to intraperitoneal vasculature or traversal of the liver capsule occurring increases when multiple passes are made through the liver for TIPS and can cause hemothorax. Excessive blood loss can also result from extrahepatic puncture of the main portal vein, causing massive hemoperitoneum after balloon dilation. These etiologies are less likely because only 100 mL of blood loss was recorded intraoperatively and correct portosystemic shunt placement was documented. Right upper quadrant ultrasound 24 hours post-procedure also showed stable ascites with no hematoma or collection at the porta hepatis. Finally, flash malignant pleural effusion is unlikely as computed tomography (CT) chest, CT abdomen and pelvis, and bronchoscopy within the month prior to admission did not show any potential sources of malignancy. 

## Conclusions

This case seeks to highlight a rare, yet serious, complication of TIPS so that preventative and supportive measures can be taken. While treating these patients, vasopressors, IV fluids, and blood infusions are essential for maintaining patient's hemodynamic stability. Close-interval chest X-ray follow-up is also important to monitor patient’s pleural effusions’ sizes and need for a thoracentesis and chest tubes. In our case, angiography was not conducted to definitely determine the cause of bleeding due to patient instability. However, given the knowledge of the procedure and anatomy, we can hypothesize that the source of bleeding was likely from bloody peritoneal fluid from the procedure, damage to the jugular vein or other blood vessels, or damage to the liver capsule. Future studies would benefit from emergent angiogram if the patient is stable enough to identify a definite source of bleeding. 
